# Awareness of Future Type 2 Diabetes Risk Among Parous Women With and Without Self-Reported Prior Gestational Diabetes in Riyadh, Saudi Arabia: A Questionnaire-Based Cross-Sectional Survey

**DOI:** 10.3390/healthcare14162572

**Published:** 2026-08-17

**Authors:** Majed Alqahtani, Fahad Alluhaydan, Khalid AlKathiri, Mohammed Alkhulayfi, Mohammed AlRabiah, Abrahem Alkhudair, Mashael Alshebaili

**Affiliations:** 1 College of Medicine, King Saud University, Riyadh 12372, Saudi Arabia; 443100304@student.ksu.edu.sa (M.A.); 443100339@student.ksu.edu.sa (F.A.); 443100093@student.ksu.edu.sa (M.A.); 443100211@student.ksu.edu.sa (M.A.);; 2 Department of Obstetrics and Gynecology, King Khalid University Hospital, College of Medicine, King Saud University, Riyadh 12372, Saudi Arabia

**Keywords:** gestational diabetes mellitus, type 2 diabetes, risk awareness, postpartum screening

## Abstract

**Background:** Gestational diabetes mellitus (GDM) is a major opportunity for type 2 diabetes mellitus (T2DM) prevention, yet gaps remain in the transition from postpartum care to long-term diabetes prevention despite clear management guidelines. This study evaluated awareness of future T2DM risk among parous women in Riyadh, Saudi Arabia, comparing women with and without a self-reported history of GDM, and identified sociodemographic and clinical determinants of risk awareness and adherence to follow-up and preventive measures. **Methods:** This observational, questionnaire-based cross-sectional survey included 316 parous women in Riyadh (*n* = 88 with self-reported prior GDM; *n* = 228 without), recruited non-probabilistically via a self-administered Google Forms questionnaire distributed through WhatsApp. GDM status, risk awareness, sociodemographic parameters, and postpartum screening and lifestyle-modification outcomes were all self-reported and not verified against medical records. Full awareness was defined a priori as selecting the highest of three ordered response options (“Yes, I am fully aware”) on a single item assessing whether GDM increases future T2DM risk; the remaining two options were combined as low/moderate awareness. Analyses used Pearson’s Chi-square tests and multivariable logistic regression to compute adjusted Odds Ratios (aOR). The primary between-group comparison was adjusted for age and educational attainment, with equivalence assessed by two one-sided tests (TOST) and subgroup differences by likelihood-ratio interaction tests. **Results:** A prior self-reported history of GDM was not associated with significantly elevated long-term risk awareness when compared with parous women without self-reported prior GDM (71.6% vs. 66.2% full awareness; *p* = 0.361, phi coefficient, φ = 0.06); this remained the case after adjustment for current age and education (aOR = 1.02, 95% CI 0.57–1.82, *p* = 0.936), and formal equivalence could not be established at a ±10 percentage-point margin (TOST *p* = 0.209). Older current age (≥45 years) was associated with higher awareness (78.0% vs. 60.3%; OR = 2.34, 95% CI 1.41–3.88, *p* = 0.0009). Within the GDM subset, structured postpartum counseling was associated with higher awareness levels (89.5% for scheduled appointments vs. 50.0% for no counseling; *p* = 0.002). In the whole sample of 316 parous women, multivariable analysis identified high awareness as the strongest independent correlate of self-reported adherence to postpartum glucose screening (aOR = 3.57, 95% CI 2.04–6.23, *p* < 0.001), with prior GDM diagnosis also independently associated with screening (aOR = 2.18, 95% CI 1.27–3.74, *p* = 0.005). Because postpartum glucose screening is specifically indicated only after GDM, a corresponding analysis was restricted to the 88 women with self-reported prior GDM, in whom 66.7% of those with full awareness reported having been screened compared with 16.0% of those with low/moderate awareness (OR = 10.50, *p* < 0.001). **Conclusions:** A prior history of GDM did not translate into higher self-reported awareness of future T2DM risk. Within the prior-GDM subgroup, structured postpartum counselling was associated with higher risk awareness, which was in turn associated with greater self-reported adherence to postpartum screening. Given the cross-sectional design and self-reported exposures and outcomes, these findings represent associations only and cannot establish causality or direction of effect.

## 1. Introduction

Gestational diabetes mellitus (GDM) is currently defined by the American Diabetes Association (ADA) as diabetes diagnosed in the second or third trimester of pregnancy that was not clearly overt diabetes prior to gestation [1]. This separates GDM from overt diabetes first detected during pregnancy, a distinction formalised in the criteria of the International Association of Diabetes and Pregnancy Study Groups (IADPSG) and adopted by the World Health Organization (WHO), which subdivides hyperglycaemia first detected in pregnancy into “diabetes mellitus in pregnancy” and the milder category of “gestational diabetes mellitus” [2,3]. Under these criteria, GDM is diagnosed on a 75-g oral glucose tolerance test (OGTT) when the fasting plasma glucose is 5.1–6.9 mmol/L, the 1-h value is ≥10.0 mmol/L, or the 2-h value is 8.5–11.0 mmol/L, whereas values reaching the non-pregnant diagnostic thresholds denote diabetes in pregnancy rather than GDM [2,3]. GDM, so defined, confers an approximately seven- to ten-fold increased risk of subsequent T2DM that persists for decades after the index pregnancy [4,5], with cumulative incidence reaching approximately 16% across study populations postpartum [6,7].

Recognizing this window for intervention, the WHO criteria described above were framed explicitly to identify women who require careful follow-up after delivery rather than discharge from surveillance [3]. The ADA further specifies that because GDM reflects pregnancy-induced insulin resistance superimposed on each woman’s underlying β-cell secretory reserve, adiposity, genetic susceptibility to type 2 diabetes, and background cardiometabolic risk, women should undergo an OGTT at 4–12 weeks postpartum, followed by lifelong screening for diabetes or prediabetes at intervals of no more than three years [8]. Implementation of these guidelines nonetheless faces persistent barriers internationally: a systematic review and Bayesian network meta-analysis of 34 studies pooling over 17,000 women reported persistently low baseline postpartum glucose-testing attendance, with only modest gains from reminder-based and educational interventions [9]. Postpartum psychosocial stressors, systemic care-coordination gaps, and limited health literacy have been identified as recurring barriers in qualitative work [10,11].

In Riyadh, Saudi Arabia, the high prevalence of T2DM and GDM has become a major public health concern [12]. This burden is regional rather than narrowly national: a systematic review and meta-analysis of 102 studies from the Middle East and North Africa estimated a pooled GDM prevalence of 13.0% (95% CI 11.5–14.6), with the highest subregional estimate in Gulf Cooperation Council (GCC) states (14.7%, 95% CI 13.0–16.5%) [13]. Contemporary Saudi evidence indicates that the postpartum care gap is at least as wide locally as it is internationally. In a three-year retrospective cohort at a tertiary centre in western Saudi Arabia, postpartum screening was ordered for 72.5% of women with GDM but completed by only 20.2%, and T2DM was already present in 13.9% of those actually screened [14]; a separate cross-sectional review at another Saudi tertiary hospital similarly documented low postpartum screening uptake and inconsistent documentation of counselling about future metabolic risk [15]. Recent Saudi surveys have identified substantial and persistent gaps in GDM-related knowledge among women, varying with maternal age, parity and educational attainment [16,17], a pattern corroborated by a more recent survey in the Northern Borders Province reporting that only 34.3% of women demonstrated good GDM awareness [18]; these studies have largely examined knowledge of GDM itself and its perinatal consequences rather than perception of the mother’s own long-term T2DM risk.

The Saudi Vision 2030 Health Transformation Program aims to shift the healthcare paradigm from reactive disease care to proactive preventive medicine, an objective that depends on detailed local data on risk awareness. While global literature highlights standalone knowledge gaps among women with a history of GDM, there remains a scarcity of regional comparative data evaluating this awareness against a comparison group of parous women drawn from the same population. Quantifying this disparity is essential to determine whether the lived experience of a GDM diagnosis effectively serves as a catalyst for health education within the local socio-demographic context.

Parous women without self-reported prior GDM served as the comparison group. This benchmark is directly interpretable for the primary outcome: because every parous woman is a candidate for primary T2DM prevention, the general parous population indicates whether a GDM diagnosis functions as a “teachable moment” conferring awareness above the population baseline. For postpartum glucose screening, however, the comparison is descriptive rather than a guideline-adherence measure, since OGTT at 4–12 weeks postpartum is specifically indicated only after GDM; the clinically interpretable adherence analysis is therefore restricted to the prior-GDM subgroup, reported separately below.

Accordingly, this study specified a single primary outcome—comparative awareness of future T2DM risk between parous women with and without a self-reported history of GDM in Riyadh—together with secondary and exploratory outcomes: (i) sociodemographic and clinical determinants of this awareness, including current age, educational attainment, and, within the prior-GDM subgroup, intensity of postpartum counselling; (ii) self-reported adherence to postpartum glucose screening; and (iii) self-reported adoption of preventive lifestyle modifications. Secondary and exploratory outcomes are interpreted as hypothesis-generating rather than co-equal confirmatory aims.

The sample-size calculation (Section 2.1) targeted precision for the overall awareness-prevalence estimate, not power for the primary between-group comparison; this is addressed further in Section 5.

## 2. Materials and Methods

### 2.1. Study Design, Setting, and Population

An observational, cross-sectional, questionnaire-based study was conducted in Riyadh, Saudi Arabia, reported in accordance with STROBE guidance for cross-sectional studies. The study targeted adult Saudi women residing in Riyadh who had been pregnant at least once. Data collection took place after IRB approval (Project No. R26-IRB-315, granted 4 May 2026), running from 5 May 2026 to 14 May 2026, within the approved study window (expiring 4 May 2027).

Participants were divided into two groups by self-reported history of GDM, based on a single questionnaire item asking whether a physician had ever diagnosed them with GDM; no verification against medical records, laboratory reports, or hospital databases was undertaken, as the study had no access to clinical data sources. The comparison group is accordingly described throughout as women without self-reported prior GDM rather than as a normoglycemic control group, since it may include women never screened during pregnancy, screened but not informed of the result, or unable to recall a prior diagnosis. Women were excluded if younger than 18 years, non-Saudi, living outside Riyadh, never pregnant, or submitting an incomplete survey.

Parity was determined by a single self-reported item (“have you ever been pregnant?”), which does not distinguish delivery outcome (live birth, miscarriage, termination) or exclude an ongoing first pregnancy; the term “parous” therefore denotes self-reported pregnancy history rather than a verified completed delivery. This is addressed as a limitation in Section 5.

Women with current prediabetes were neither identified nor excluded, and could be present in either group. As with pre-existing T2DM, a prediabetes diagnosis would plausibly increase both risk awareness and screening adherence independently of a prior GDM diagnosis; this is addressed alongside the T2DM caveat in Section 5.

The required sample size was calculated using a single-proportion formula (95% confidence level, 5% margin of error, assumed baseline awareness prevalence of 50% to maximize sample size), giving a minimum of 384 participants, increased by 20% for non-response to a target of 461. This calculation addresses the precision of a prevalence estimate rather than power for the primary between-group comparison; a two-proportion calculation would have required a substantially larger sample (Section 5).

**Figure 1:**
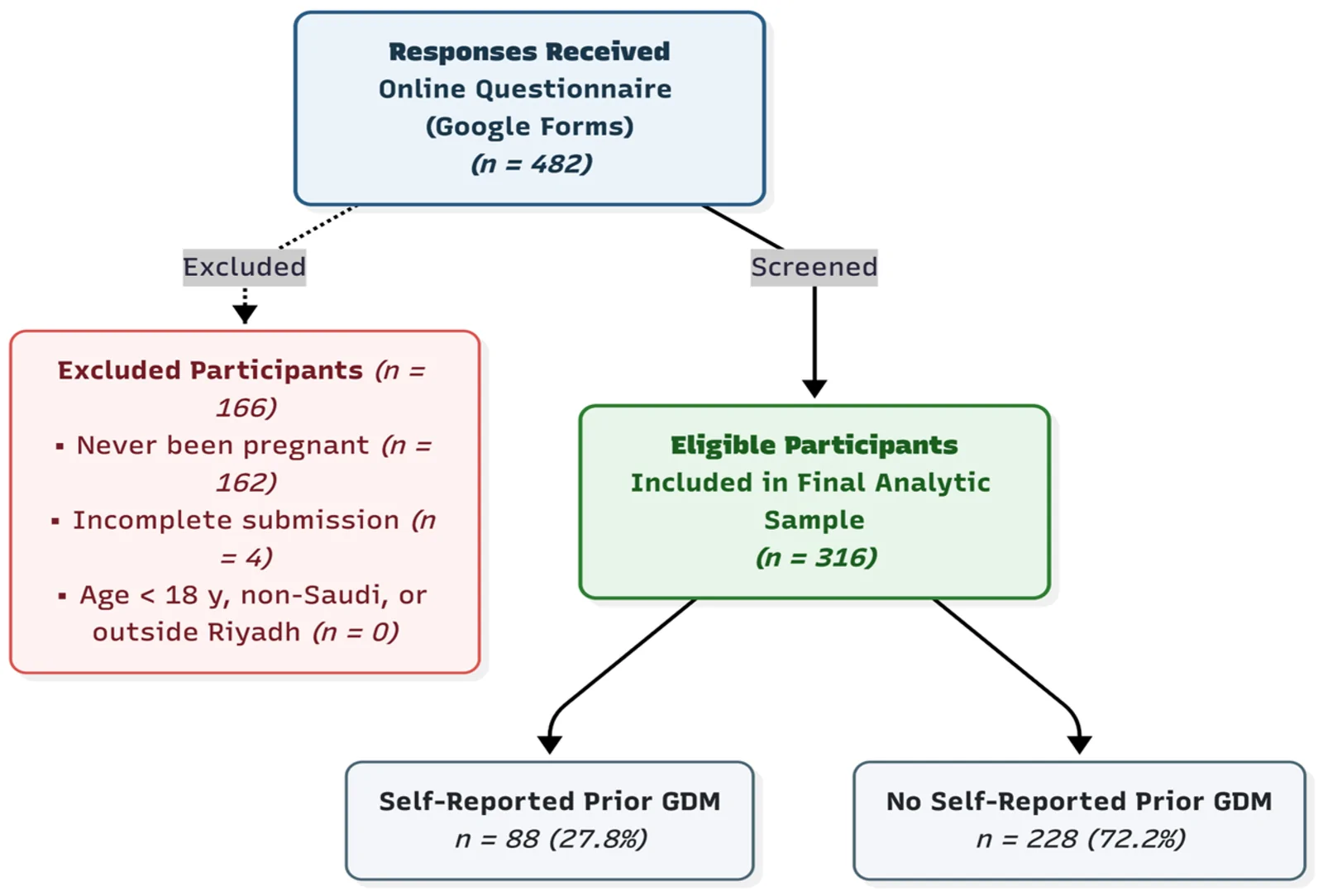
Participant flow diagram. Of 482 responses received, 162 were excluded for never having been pregnant and 4 for incomplete submission (166 total exclusions); no respondent was excluded for age under 18 years, non-Saudi nationality, or residence outside Riyadh (Section 2.1). The final analytic sample comprised 316 women, of whom 88 (27.8%) self-reported prior GDM and 228 (72.2%) did not.

In total, 482 responses were collected; 162 respondents who had never been pregnant and 4 incomplete submissions were excluded, leaving 316 eligible participants (Figure 1). No respondent was excluded for age under 18, non-Saudi nationality, or residence outside Riyadh, as the questionnaire did not include a separate screening item for these criteria independent of eligibility to proceed. All items were mandatory except current weight and height (Section 2.2); item-level missingness was therefore confined to these optional anthropometric fields and did not reduce the analytic denominator. The denominator for full-sample analyses is *n* = 316; for analyses restricted to women with self-reported prior GDM, *n* = 88; for the comparison group, *n* = 228, unless otherwise stated. The response options “I do not remember” and “I was not asked to” on the postpartum screening item (Section 2.3) are substantive response categories, coded as non-adherence in the primary analysis.

### 2.2. Data Collection Protocols and Variables

Data were collected using a 22-item anonymous online survey structured via Google Forms, reported with reference to CHERRIES guidance for internet e-surveys. The survey link was distributed exclusively via WhatsApp to reach a diverse audience across Riyadh. Recruitment was accordingly online and non-probabilistic, following a convenience sampling approach; no clinic-based, registry-based, or population-based sampling frame was used, and no response rate could be calculated for an openly distributed link.

The questionnaire was originally drafted in English. The Arabic version was reviewed for content validity by an Associate Professor and Consultant in Obstetrics and Gynecology, and both language versions were reviewed by field experts for content validity, clarity, and relevance to the study objectives; minor wording adjustments were made based on this feedback. A pilot study was conducted with 10 eligible participants prior to formal data collection to assess questionnaire clarity and feasibility. The questionnaire was administered bilingually: matched English- and Arabic-language versions (Supplementary Files S1 and S2) were both made available, and respondents could choose either.

Because the survey collected no personal identifiers and did not require respondent sign-in (Section 2.4), Google Forms’ native “limit to one response” feature could not be enabled; duplicate submissions were instead evaluated during data cleaning by inspecting submission timestamps and demographic profiles. The interface allowed respondents to navigate back and revise answers before final submission; once submitted, responses could not be retrieved or edited, consistent with the anonymous design (Section 2.4).

The primary dependent variable was participants’ self-reported awareness that gestational diabetes increases a woman’s risk of developing type 2 diabetes later in life, referred to throughout as risk awareness—general awareness of the GDM–T2DM association rather than a personalised assessment of individual risk, which was not separately measured. This was captured by a single item (“Are you aware that having gestational diabetes increases a woman’s risk of developing Type 2 Diabetes later in life?”) offering three ordered response options. “Yes, I am fully aware” was classified as full (high) awareness; “I have heard about it, but I’m not sure” and “No, I was not aware” were combined into a low/moderate awareness category. This dichotomisation was specified before analysis; the full three-level variable was retained for the ordinal sensitivity analysis (Section 2.3). No composite score was computed, and no other item was combined with this one to derive the awareness variable.

The dichotomisation threshold was chosen because only “Yes, I am fully aware” represents unambiguous, unqualified awareness, while “I have heard about it, but I’m not sure” signals uncertainty judged clinically closer to non-awareness for the purpose of predicting protective health behaviour, consistent with Health Belief Model formulations in which unambiguous perceived susceptibility, rather than vague familiarity, is the construct expected to motivate action. Since this is one of several defensible cut-points, the full three-level variable was additionally modelled without dichotomisation via proportional-odds ordinal logistic regression (Section 2.3); closely similar estimates from the ordinal model indicate the conclusions are not an artefact of this threshold. For clarity, this manuscript uses “risk awareness” specifically and consistently to denote responses to this single item; “knowledge” and “health literacy” are used only when describing constructs measured in cited literature, since no validated knowledge test or health-literacy instrument was administered here.

Age was ascertained as current age at survey completion (a single categorical item: 18–24, 25–34, 35–44, or ≥45 years); the oldest category was open-ended by questionnaire design, so all statistics involving age are based on these four fixed bands rather than a continuous measure, and age is referred to throughout as current age rather than maternal age. Independent variables included history of gestational diabetes, current age, highest level of education, and current employment status. Employment status (item 5: employed, homemaker/unemployed, or student) is reported descriptively in Section 3.1 but was not entered as a covariate in any regression model. The questionnaire additionally asked, among women with prior GDM, how gestational diabetes had been managed during the index pregnancy (dietary/lifestyle modification only versus oral medication or insulin; item 10), and asked all respondents a hypothetical item on the main perceived barrier to a healthy lifestyle, had they not been adherent to one (item 22); these items are reported descriptively in Section 3.1 but were not entered as covariates. The questionnaire (Supplementary Files S1 and S2) does not include an item on gravidity or parity. Time since the most recent delivery was not collected as a general item for the whole sample; only women reporting prior GDM were asked how long it had been since the GDM-affected pregnancy (item 9).

Within the prior-GDM subgroup, intensity of postpartum counselling was ascertained from a single item asking whether the respondent had been “advised to undergo regular diabetes screening after delivery,” with three response options: “Yes, and follow-up appointments were scheduled” (formally scheduled appointments), “Yes, but only through verbal advice” (verbal counselling only), and “No, I was not advised” (no structured counselling). The item did not specify a timing window, so responses may reflect advice received at any point from the index pregnancy onward. “Formally scheduled appointments” denotes that a follow-up appointment or referral date had been arranged; it does not, by itself, confirm attendance or screening completion, which is captured separately by the postpartum screening item below.

The postpartum screening outcome was likewise a single self-reported item: “Did you get your blood sugar checked 6 to 12 weeks after you delivered your baby?” (Yes/No/I don’t remember/I wasn’t asked to do that), with “Yes” coded as adherent and all other responses coded as non-adherent in the primary analysis (Section 2.3). The item asks generically whether blood glucose was checked and does not specify the test used (e.g., a 75-g OGTT, fasting plasma glucose, random glucose, or HbA1c); the outcome should be read as any self-reported postpartum glucose testing rather than guideline-specific OGTT completion. The survey’s 6–12-week window is also narrower than the 4–12-week window in the ADA guidance cited in the Introduction; item wording was fixed at survey design and was not re-derived from the ADA window, so the two should not be read as equivalent.

Adoption of preventive lifestyle modification was ascertained from a multi-select item asking which changes the respondent had made: improving diet, increasing physical activity/exercise, actively managing weight, or none of the above. Respondents selecting at least one of the first three options were classified as having adopted a lifestyle modification; those selecting only “No changes made” were classified as not having done so. This at-least-one-endorsement rule—selection of at least one specified change, rather than a count of endorsements—was applied throughout the analysis.

The clinical meaning of postpartum screening adherence differs by group: among women with self-reported prior GDM, a postpartum glucose check is the guideline-indicated procedure described in the Introduction, whereas among women without self-reported prior GDM, the same item captures opportunistic or incidental glucose testing rather than adherence to a specific guideline. This distinction motivates treating the whole-sample regression as descriptive of correlates of glucose testing in general, and the analysis restricted to the prior-GDM subgroup as the clinically interpretable adherence estimate.

### 2.3. Statistical Framework

Statistical analyses were performed using SPSS software (version 31.0.0; IBM Inc., Chicago, IL, USA). Descriptive statistics were computed to summarize the continuous and categorical baseline traits of the participants. Categorical variables are presented as counts and percentages with 95% Wilson score confidence intervals.

To compare the groups and evaluate how their awareness and specific clinical outcomes associated, Pearson’s Chi-square tests of independence were applied, with the Fisher–Freeman–Halton exact test substituted where more than 20% of cells had expected counts below five. Cramer’s V was calculated to measure the strength of those associations; for 2 × 2 tables, the phi coefficient is reported instead. This expected-cell-count threshold was checked for every cross-tabulation reported in the main text and in tables; none exceeded the threshold, so standard Pearson chi-square statistics are reported throughout. Because the two comparison groups differed substantially in age distribution, the primary between-group comparison of risk awareness was additionally examined in a multivariable logistic regression model adjusted for current age and educational attainment. As a sensitivity analysis, the three-level awareness variable was modelled using proportional-odds ordinal logistic regression to avoid information loss from dichotomisation. Because the study’s principal comparative finding was a null result, two one-sided tests (TOST) were conducted against a pre-specified equivalence margin of ±10 percentage points to determine whether statistical equivalence could be formally established.

We then built a multivariable logistic regression model to identify factors independently associated with screening adherence after adjustment for selected covariates. This model adjusted for history of gestational diabetes, age group, and educational attainment, generating adjusted Odds Ratios (aOR) and 95% Confidence Intervals. Model adequacy was assessed using the Hosmer–Lemeshow goodness-of-fit test, the C-statistic, variance inflation factors, and the events-per-variable ratio. Subgroup differences were evaluated by formal likelihood-ratio tests of interaction rather than by comparison of subgroup-specific *p*-values. Because 31.0% of respondents answered the postpartum screening item with “I do not remember” or “I was not asked to,” these responses were coded as non-adherent in the primary analysis, and a pre-specified sensitivity analysis re-estimated all models with these responses excluded.

The Benjamini–Hochberg procedure was applied to control the false discovery rate across all reported hypothesis tests, spanning the primary outcome (risk awareness) and the secondary/exploratory outcomes (postpartum screening and lifestyle modification, and their sociodemographic and clinical correlates); lists this correction alongside the other sensitivity analyses. Some residual risk of false-positive findings remains given the number of comparisons performed. For all tests, statistical significance was set at a *p*-value of 0.05 or lower.

### 2.4. Ethical Considerations

This study was approved by the Institutional Review Board (IRB) at King Saud University (Project No. R26-IRB-315, Ref. No. 26/0345/IRB; approved 4 May 2026, expiring 4 May 2027). Electronic informed consent was obtained from all participants prior to accessing the questionnaire. The introductory page presented a brief information sheet describing the study’s purpose, approximate completion time (3–5 min), and an assurance that participation was voluntary and responses anonymous and confidential; respondents were then asked: ‘Do you consent to participate in this study?’ with response options ‘Yes (Proceed to survey)’ and ‘No (End survey)’. Only respondents selecting ‘Yes’ proceeded to substantive questionnaire items. Because the survey was anonymous, participants could decline to continue at any point before submitting; once submitted, a response could not be withdrawn, as no identifying information was collected that would allow it to be located and removed.

## 3. Results

### 3.1. Baseline Sociodemographic Characteristics

The final analytical sample consists of 316 parous women. Stratification by self-reported clinical history revealed 88 women (27.8%) reporting a previous physician diagnosis of GDM, and a larger comparison group of 228 women (72.2%) without self-reported prior GDM. The prior GDM group was significantly advanced in age, accounting for 61.4% of participants aged 45 years or older, compared to only 34.2% within the no-prior-GDM group (*p* < 0.001). Educational profiles also varied (*p* = 0.021), although obtaining a bachelor’s degree remained the majority case in both groups (Table 1). Overall, 231 of the 316 participants (73.1%) held a bachelor’s degree or higher (Table 1), a considerably more educated profile than the general population of parous women in Riyadh. Because educational attainment is a plausible correlate of general health engagement and survey participation, the awareness estimates reported here should be interpreted as an upper bound for, rather than as directly generalisable to, the wider population of parous women in the city; this is discussed further in Section 5. Regarding employment status, 159 women (50.3%) were employed, 153 (48.4%) were homemakers or not currently employed, and 4 (1.3%) were students; stratified counts by prior GDM status were not available.

**Table 1:** Baseline Sociodemographic Characteristics Stratified by Prior GDM Status.

Characteristic	Total Sample (N = 316)	Prior GDM (*n* = 88)	No-Prior GDM (*n* = 228)	*p*-Value
Age Group				<0.001
18–24 years	6 (1.9%)	1 (1.1%)	5 (2.2%)	
25–34 years	77 (24.4%)	8 (9.1%)	69 (30.3%)	
35–44 years	101 (32.0%)	25 (28.4%)	76 (33.3%)	
≥45 years	132 (41.8%)	54 (61.4%)	78 (34.2%)	
Educational Attainment				0.021
High School or less	53 (16.8%)	23 (26.1%)	30 (13.2%)	
Diploma	32 (10.1%)	11 (12.5%)	21 (9.2%)	
Bachelor’s Degree	195 (61.7%)	47 (53.4%)	148 (64.9%)	
Postgraduate	36 (11.4%)	7 (8.0%)	29 (12.7%)	

Note. The ≥45 years category was an open-ended top band at questionnaire design (Section 2.2), so no exact maximum participant age can be reported. Age counts are derived exactly from the percentages and marginal totals reported in Section 3.1 and Table 2 (e.g., 61.4% of 88 = 54; totals of 132 and 184 across the whole sample match Table 2 exactly). Exact cell counts for employment status, stratified by prior GDM status, were not available in the summary output prepared for this manuscript; only the unstratified totals are reported in the text (Section 3.1). Body mass index is not reported here because of unresolved unit-conversion inconsistencies in the anthropometric data (Section 5).

**Table 2:** Bivariate Analysis of Factors Associated with High T2DM Risk Awareness.

Predictor	High Awareness *n*/N	% (95% CI)	Effect Estimate	*p*-Value
GDM history				
Prior GDM	63/88	71.6 (61.4–80.0)	OR 1.29 (0.75–2.20)	0.361
No prior GDM	151/228	66.2 (59.9–72.1)	Reference	
Current age				
≥45 years	103/132	78.0 (70.2–84.3)	OR 2.34 (1.41–3.88)	0.001
<45 years	111/184	60.3 (53.1–67.1)	Reference	
Educational attainment				
Bachelor’s degree or above	156/231	67.5 (61.2–73.3)	OR 0.97 (0.57–1.65)	0.906
Below bachelor’s degree	58/85	68.2 (57.7–77.2)	Reference	
Postpartum clinical counselling (prior GDM subset, *n* = 88)				
Formally scheduled appointments	34/38	89.5 (75.9–95.8)	OR 8.50 (2.34–30.9)	0.002
Verbal counselling only	16/24	66.7 (46.7–82.0)	OR 2.00 (0.64–6.29)	
No structured counselling	13/26	50.0 (32.1–67.9)	Reference	

Note. Proportions are shown with 95% Wilson score confidence intervals. Each *p*-value corresponds to the dichotomous contrast displayed. The counselling omnibus test gives χ^2^ = 12.22, df = 2, *p* = 0.0022, Cramer’s V = 0.373, with a significant test for linear trend (OR = 2.87 per level, 95% CI 1.50–5.49, *p* = 0.0014).

Family history of type 2 diabetes was ascertained only among women with prior GDM (Section 2.2) and cannot be stratified by comparison-group status. Time since most recent delivery was not collected for the whole sample; time since the GDM-affected pregnancy, collected for the prior-GDM subgroup only, is reported separately below.

**Figure 2:**
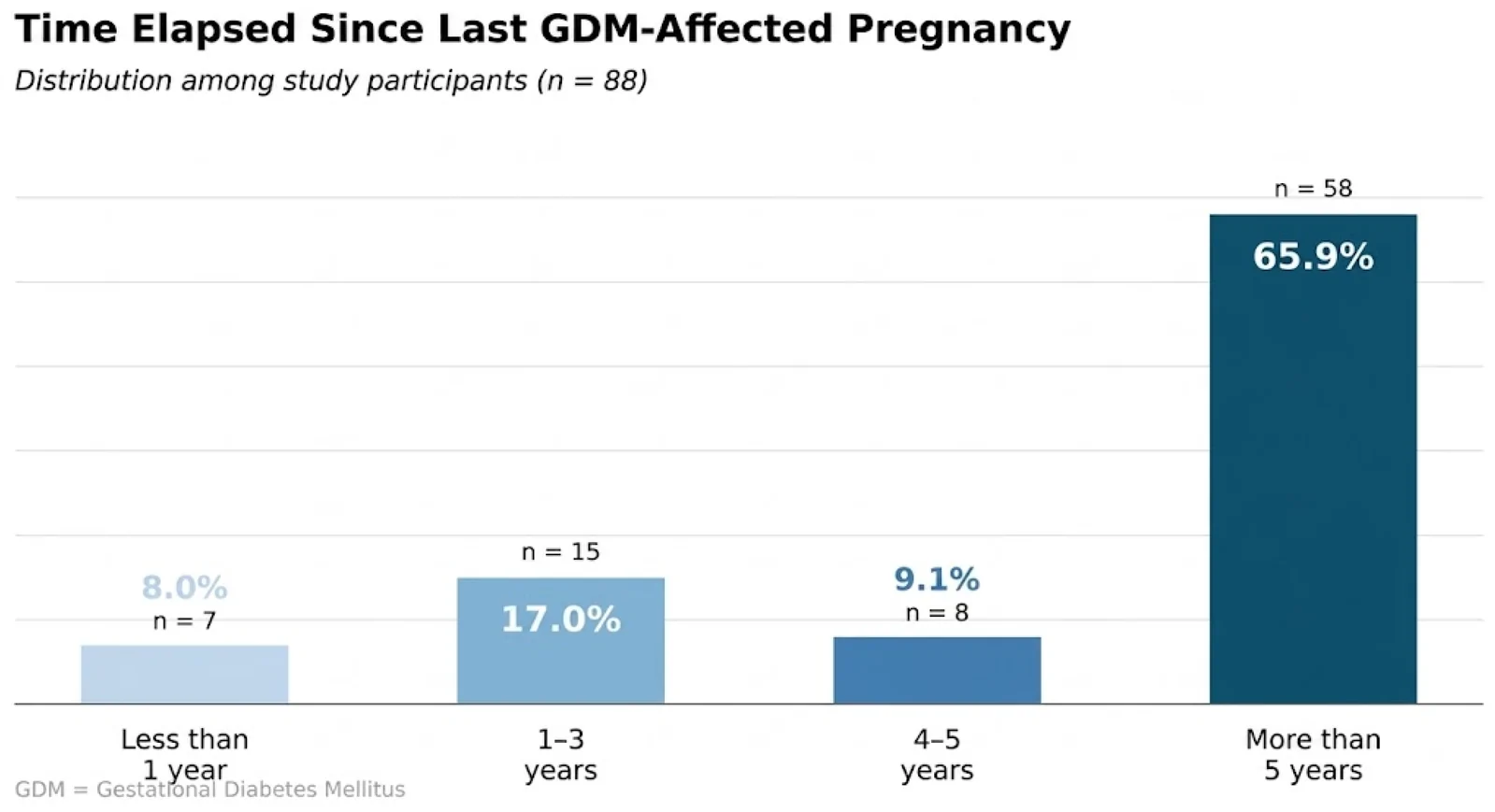
Time elapsed since the last GDM-affected pregnancy among women with self-reported prior GDM (*n* = 88). Distribution: less than 1 year, 7/88 (8.0%); 1–3 years, 15/88 (17.0%); 4–5 years, 8/88 (9.1%); more than 5 years, 58/88 (65.9%).

**Figure 3:**
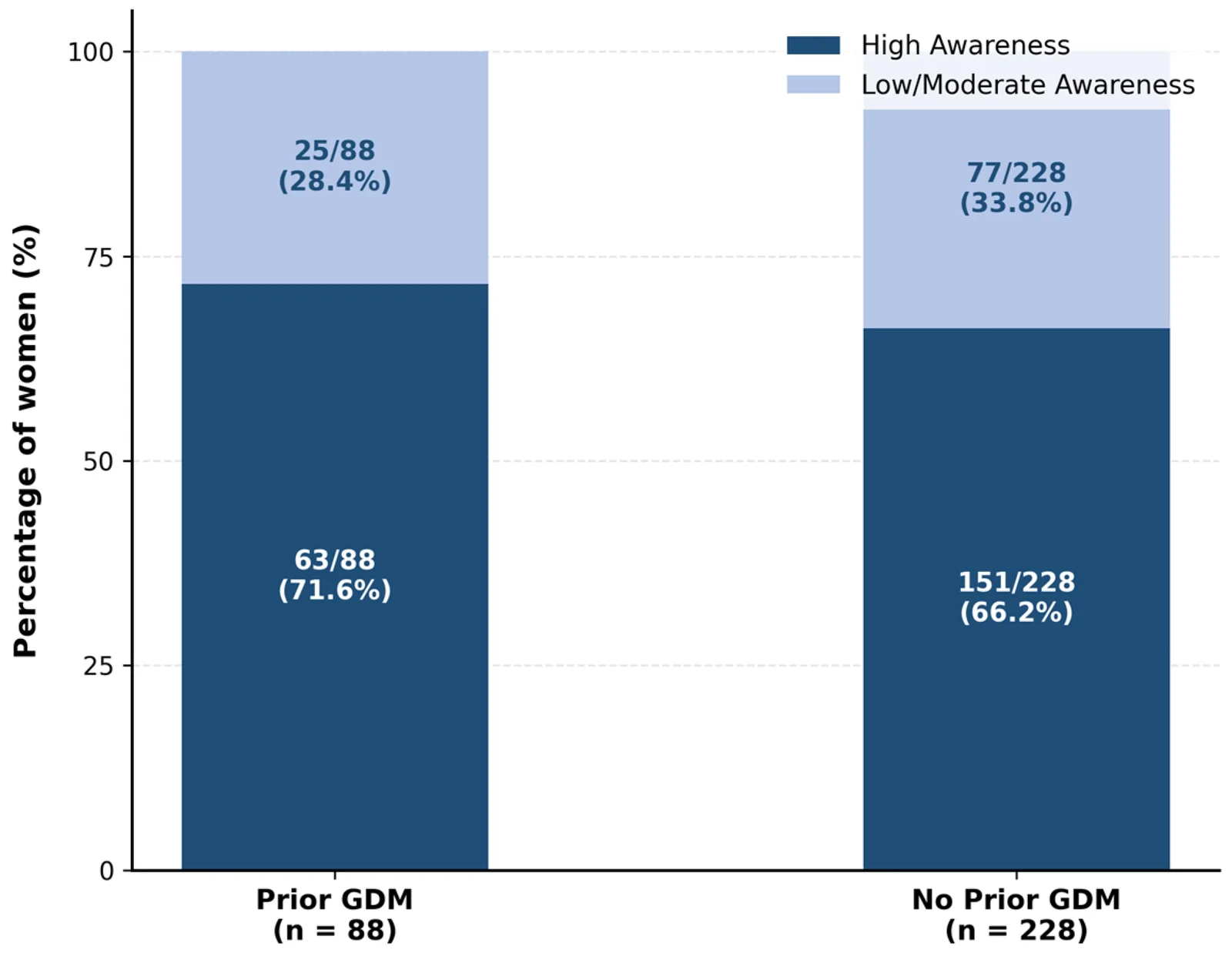
Self-reported awareness of future T2DM risk (High Awareness versus Low/Moderate Awareness; Section 2.2), stratified by prior GDM status. Prior GDM: 63/88 (71.6%) high awareness, 25/88 (28.4%) low/moderate awareness. No prior GDM: 151/228 (66.2%) high awareness, 77/228 (33.8%) low/moderate awareness. These figures are reconciled with, and identical to, Table 2.

Among the 88 women with self-reported prior GDM, time since the GDM-affected pregnancy (Section 2.2, item 9) is shown in Figure 2. This variable was not included as a covariate in the multivariable models reported in Section 3.3 and Section 3.4, because awareness and screening behaviour would plausibly change with time elapsed since the index pregnancy; this omission is addressed as a limitation in Section 5. Among these 88 women, the index pregnancy had been managed with diet/lifestyle modification alone in 59 (67.0%), with oral medication or insulin in 20 (22.7%), and 9 (10.2%) did not remember how it had been managed (Figure 3).

### 3.2. Comparison of T2DM Risk Awareness by Prior GDM Status

The primary objective was to evaluate the comparative awareness of future T2DM risk. At the overall level, self-reported awareness was strong across the sample. Within the Prior GDM group, 71.6% (63/88, 95% CI 61.4–80.0) of women reported that they were “fully aware” of the escalating T2DM risk in the future. In parallel, 66.2% (151/228, 95% CI 59.9–72.1) of women without self-reported prior GDM similarly reported full awareness of future T2DM risk. A Chi-square test indicated that this observed margin was not statistically significant (*p* = 0.361), resulting in a negligible effect size (phi coefficient, φ = 0.06); the absolute difference between groups was +5.4 percentage points (95% CI −5.9 to +16.6). Because the prior GDM group was substantially older than the comparison group (61.4% versus 34.2% aged ≥ 45 years) and age was itself strongly associated with awareness, this crude comparison was repeated with adjustment for current age and educational attainment. After adjustment, prior GDM showed no independent association with high risk awareness (aOR = 1.02, 95% CI 0.57–1.82, *p* = 0.936), and a proportional-odds ordinal model using all three awareness categories yielded a closely similar estimate (OR = 0.97, 95% CI 0.55–1.72, *p* = 0.929). The crude difference observed between groups was therefore attenuated after adjustment for current age, consistent with the unadjusted contrast reflecting the age imbalance between groups rather than an independent effect of the GDM diagnosis itself. These findings indicate that no difference in risk awareness was detected between the two groups. They should not, however, be interpreted as demonstrating equivalence. Formal two one-sided tests against a ±10 percentage-point margin did not establish equivalence (*p* = 0.209), and with 88 women in the prior GDM group the study had 80% power to detect only differences of approximately 15 percentage points or greater. A smaller but clinically meaningful advantage for women with prior GDM cannot be excluded on the basis of these data.

### 3.3. Sociodemographic and Clinical Correlates of Risk Awareness

To identify factors associated with higher risk awareness, participants were classified into binary groups of “High Awareness” versus “Low/Moderate Awareness” (abbreviated “Low/Mod Awareness”, defined there in a footnote). An older current age (≥45 years) was a statistically significant positive indicator, with 78.0% (95% CI 70.2–84.3) of women in this category showing high awareness, compared with 60.3% (95% CI 53.1–67.1) of younger women (OR = 2.34, 95% CI 1.41–3.88, *p* = 0.0009). Conversely, educational attainment showed no significant association with risk awareness (67.5% for bachelor’s degree or above versus 68.2% below; OR = 0.97, 95% CI 0.57–1.65, *p* = 0.906).

Within the prior-GDM subgroup, structured postpartum counselling was associated with higher awareness. Women who received formally scheduled postpartum screening appointments reported high, though not universal, awareness (89.5%), compared with 66.7% among women who received only verbal counselling and 50.0% among those receiving no structured counselling (*p* = 0.002, Cramer’s V = 0.37). A test for linear trend across increasing counselling intensity was significant (OR = 2.87 per level, 95% CI 1.50–5.49, *p* = 0.0014), indicating a graded dose–response relationship between the formality of postpartum counselling and subsequent risk awareness. This association persisted after adjustment for current age (aOR for formally scheduled appointments versus no counselling = 8.50, 95% CI 2.34–30.9, *p* = 0.002). This analysis is observational and non-randomised: women were not randomly assigned to a counselling intensity, and receipt of formally scheduled postpartum appointments may itself be a marker of, or be confounded by, differential healthcare access, continuity of provider, or overall quality of antenatal and postpartum care. The association should therefore be interpreted as hypothesis-generating rather than as evidence that scheduling appointments alone causally produces higher awareness (Table 3).

### 3.4. From Awareness to Action: Association Between Risk Awareness and Self-Reported Postpartum Screening Completion and Lifestyle Modification

This section examines self-reported adherence to postpartum glucose screening and to targeted lifestyle modifications, and its relationship to risk awareness.

Multivariable logistic regression identified high risk awareness as the strongest independent correlate of adherence to the screening across the overall sample (aOR = 3.57, 95% CI 2.04–6.23, *p* < 0.001), with prior GDM diagnosis also independently associated with screening adherence (aOR = 2.18, 95% CI 1.27–3.74, *p* = 0.005) (Table 4). Within the prior GDM subgroup, 66.7% of highly aware women complied with screening, compared with only 16.0% of those with low/moderate awareness (OR = 10.50, *p* < 0.001). The model showed adequate calibration and discrimination with no meaningful collinearity (Table 4 note). These diagnostics describe the screening-adherence model only and are separate from the power considerations for the primary risk-awareness comparison, discussed in Section 5. A pre-specified sensitivity analysis excluding respondents who could not recall their screening status yielded a comparable and slightly stronger estimate (aOR = 3.96, 95% CI 2.11–7.43, *p* < 0.001). Because risk awareness was measured concurrently with the survey while postpartum screening had occurred at an earlier time point, the direction of this association cannot be established from the present design; undergoing postpartum screening may itself have generated awareness. The relationship is therefore reported as an association rather than as a predictive or causal effect.

Secondary analysis focusing on the adoption of proactive lifestyle modifications (e.g., active weight management, dietary blood sugar control) showed that highly aware women reported higher adoption than their counterparts (82.7% versus 64.7% of adoption, *p* < 0.001). Although this association reached significance among women without self-reported prior GDM (*p* < 0.001) but not among those with prior GDM (*p* = 0.821), a formal likelihood-ratio test of the awareness × GDM interaction was not statistically significant (χ^2^ = 1.84, df = 1, *p* = 0.175), indicating that the apparent subgroup difference is not statistically supported and most plausibly reflects the smaller size of the GDM stratum. By contrast, a corresponding interaction test for the screening outcome was significant (χ^2^ = 5.15, df = 1, *p* = 0.023), suggesting that the association between awareness and screening adherence was stronger among women with prior GDM (Table 5). When asked hypothetically what would be the main barrier to a healthy lifestyle if they were not adherent to one (item 22), the most commonly cited barrier across the whole sample was lack of time (185/316, 58.5%), followed by the financial cost of healthy food or gym access (78/316, 24.7%), not being convinced of the benefit of lifestyle change (30/316, 9.5%), and lack of family support (23/316, 7.3%) (Table 6).

**Table 5:** Self-Reported Postpartum Screening Completion and Lifestyle Modification Outcomes Stratified by Risk Awareness Level and GDM History.

Outcome and Cohort	High Awareness	Low/Mod Awareness	Odds Ratio (95% CI)	*p*-Value
Completed Postpartum Screening (6–12 Weeks)				
Overall Sample (N = 316)	103 (48.1%)	21 (20.6%)	3.58 (2.05–6.25)	<0.001
Prior GDM (*n* = 88)	42 (66.7%)	4 (16.0%)	10.50 (2.98–36.96)	<0.001
No Prior GDM (*n* = 228)	61 (40.4%)	17 (22.1%)	2.39 (1.27–4.51)	0.009
Adopted Active Lifestyle Modifications				
Overall Sample (N = 316)	177 (82.7%)	66 (64.7%)	2.60 (1.49–4.53)	<0.001
Prior GDM (*n* = 88)	51 (81.0%)	19 (76.0%)	1.34 (0.42–4.31)	0.821
No Prior GDM (*n* = 228)	126 (83.4%)	47 (61.0%)	3.21 (1.68–6.13)	<0.001

Note. “Low/Mod Awareness” denotes low/moderate awareness, i.e., the combined reference category comprising the two lower response options on the awareness item (Section 2.2). All odds ratios shown in this table are crude (unadjusted), calculated directly from the stratum-specific 2 × 2 tables of high versus low/moderate awareness by adherence status; they are not adjusted for current age, education, or other covariates. Adjusted estimates for the corresponding whole-sample screening model are reported separately in Table 4 and Figure 4.

**Figure 4:**
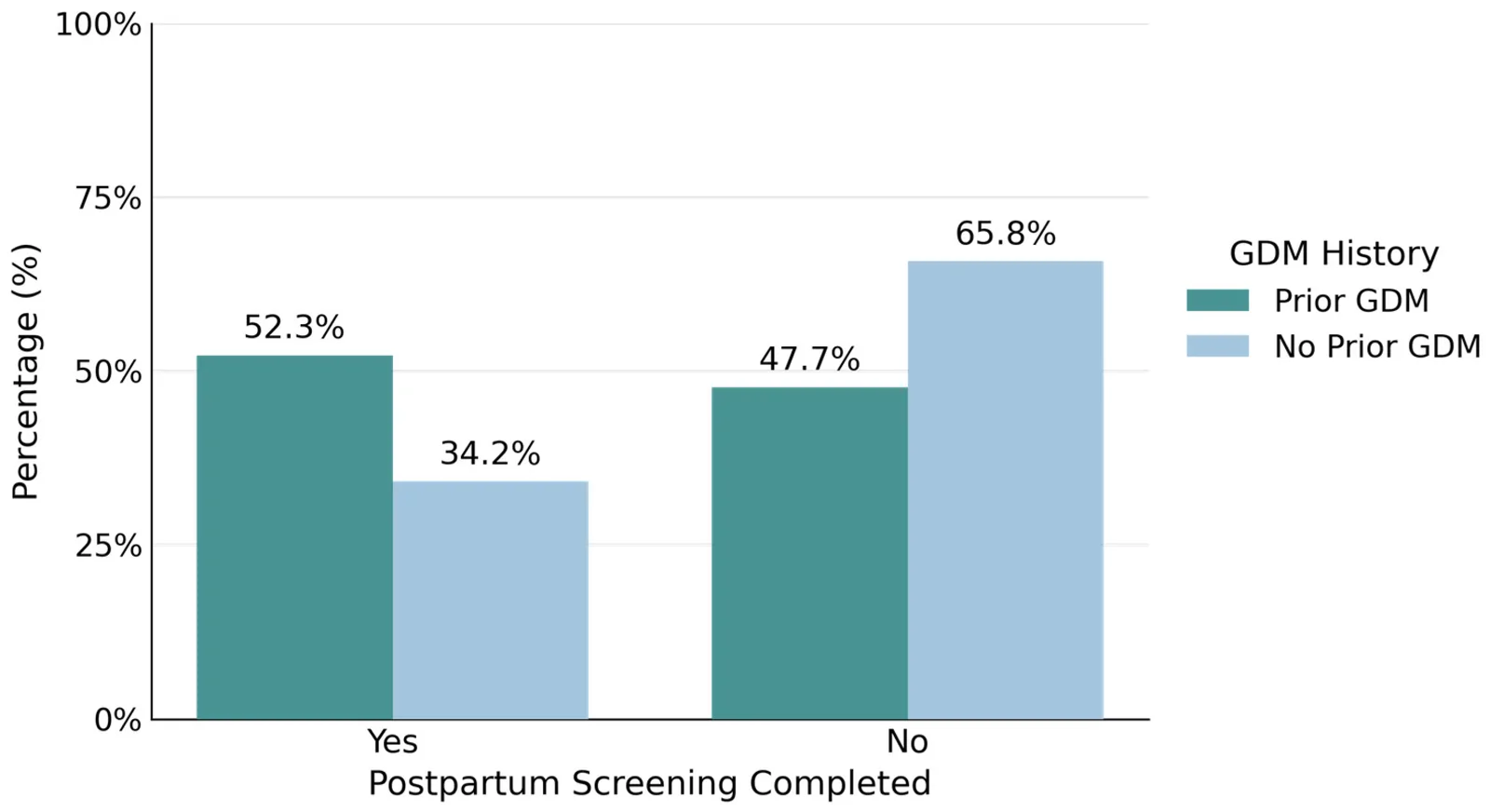
Proportion of postpartum glucose screening completion among study participants, stratified by gestational diabetes mellitus (GDM) history. Exact numerators and denominators are as follows: prior GDM, 46/88 (52.3%) screened, 42/88 (47.7%) not screened; no prior GDM, 78/228 (34.2%) screened, 150/228 (65.8%) not screened. These figures are reconciled with, and identical to, the corresponding cell counts in Table 5.

**Table 6:** Sensitivity and Robustness Analyses.

Analysis	Result	Conclusions
Primary comparison, adjusted for age and education	aOR 1.02 (0.57–1.82), *p* = 0.936	Null result confirmed
Ordinal (proportional-odds) awareness model	OR 0.97 (0.55–1.72), *p* = 0.929	Robust to dichotomisation
TOST equivalence test, ±10 pp margin	*p* = 0.209	Equivalence not established
Screening outcome recoded (ambiguous responses excluded)	Awareness aOR 3.96 (2.11–7.43)	Robust; effect strengthens
Awareness × GDM interaction, lifestyle outcome	χ^2^ = 1.84, df = 1, *p* = 0.175	No effect modification
Awareness × GDM interaction, screening outcome	χ^2^ = 5.15, df = 1, *p* = 0.023	Effect modification present
Benjamini–Hochberg FDR across 12 tests	All significant findings retained	Robust to multiplicity

## 4. Discussion

The primary objective of this study was to evaluate the baseline level of T2DM risk awareness among women in Riyadh, specifically contrasting those with a self-reported history of GDM against parous women without self-reported prior GDM. Furthermore, this investigation sought to examine the association between self-reported risk awareness and self-reported adherence to postpartum screening and lifestyle modification.

The self-reported history of GDM did not inherently amplify long-term self-reported awareness of future T2DM risk. Specifically, 71.6% of women in the prior GDM group reported full awareness that gestational diabetes increases a woman’s risk of developing type 2 diabetes later in life, a proportion that did not significantly differ from the 66.2% observed among women without self-reported prior GDM (*p* = 0.361). This absence of a detected difference, supported by a negligible effect size (phi coefficient, φ = 0.06) and unchanged after adjustment for current age and education (aOR = 1.02, 95% CI 0.57–1.82), stands in contrast to conventional clinical expectations, although the present sample was not powered to establish formal equivalence.

However, because awareness was assessed at a single cross-sectional time point rather than longitudinally, our data cannot establish whether awareness changes, or is lost, over the postpartum period; what the present data show is that prior GDM was not associated with significantly higher awareness of future T2DM risk at the time of survey. This finding aligned with several regional investigations documenting that postpartum women frequently experience a rapid decline in health surveillance once immediate neonatal risks subside [8,19], a decline plausibly driven by the intense physical and emotional demands of the postpartum period combined with poor health communication during pregnancy—where clinical discussions often emphasize immediate fetal risks over the mother’s long-term metabolic threat, leading many women to internalize the birth as a ‘cure’ for their hyperglycemia and diminishing the perceived urgency for postpartum screening [10,20,21]. The similarity in awareness between women with and without self-reported prior GDM suggests that the risk awareness regarding T2DM is a pervasive challenge that transcends the specific experience of pregnancy complications and is shared across the entire population of women in Riyadh [22,23]. Several methodological differences from prior studies likely contribute to the divergence between our results and previously reported awareness and screening estimates: differing postpartum assessment windows (our 6–12-week screening item versus longer follow-up periods or the ADA’s 4–12-week guidance), a broader definition of screening (any self-reported glucose check, rather than confirmed OGTT completion), a community-recruited social-media sample rather than clinic- or tertiary-hospital cohorts, unverified rather than clinically confirmed GDM diagnoses, use of a single risk-awareness item rather than multi-item knowledge instruments, and a more highly educated sample than the general population of parous women in Riyadh (Section 3.1). These differences should be considered when generalising our findings.

Risk awareness should also extend beyond glucose intolerance, since prior GDM is strongly associated with later hypertension and ischemic heart disease [24]. In exploring the sociodemographic determinants of risk awareness, an older current age (45 years or older) emerged as a statistically significant correlate of high risk awareness (OR = 2.34, 95% CI 1.41–3.88, *p* = 0.0009). This phenomenon could be explained by an increased lifetime exposure to public health campaigns, greater personal interaction with healthcare systems, or the gradual onset of age-related metabolic changes that naturally prompt closer health monitoring. Conversely, educational attainment demonstrated no statistically significant association with risk awareness (OR = 0.97, 95% CI 0.57–1.65, *p* = 0.906), underscoring the urgent need for targeted, plain-language clinical counseling that transcends general education levels. Beyond individual awareness, our findings also underscore the structural limitations of the healthcare system. This system fragmentation likely contributes to the lower screening rates we found, as the steps for transitioning care between providers are not well-defined [25]. Cultural and social factors—for example, traditional eating habits and beliefs about body weight documented in the broader literature [26]—may plausibly also conflict with standard clinical advice for preventing diabetes in this setting. However, the present questionnaire did not directly assess dietary beliefs or attitudes toward body weight among our own participants, so we cannot confirm that such beliefs contributed to the awareness or adherence patterns observed here; this explanation should therefore be regarded as a possible contextual factor warranting dedicated future study rather than as a finding of the present data. Adherence to GDM screening and postpartum follow-up recommendations is inconsistent across health systems, with provider-, client- and system-level factors—rather than any single determinant—shaping compliance [27].

The principal finding from our multivariable logistic regression model was that, across the entire sample of 316 parous women, high risk awareness was the strongest independent correlate of self-reported postpartum glucose screening completion (aOR = 3.57, 95% CI 2.04–6.23, *p* < 0.001). This large disparity is consistent with the perceived-susceptibility component of health behaviour frameworks such as the Health Belief Model, which posits that perceiving oneself to be at risk is an important precondition for preventive action. We did not, however, measure perceived severity, perceived benefits, perceived barriers, cues to action, or self-efficacy, and our findings should therefore not be read as a validation of the Health Belief Model as a whole, but only as consistent with its perceived-susceptibility component. Our results indicate that while a historical diagnosis of GDM alone is insufficient to trigger action, the internalization of that risk is strongly associated with returning for essential metabolic follow-up. Because awareness was ascertained after screening had occurred, however, the direction of this association cannot be determined from the present cross-sectional design, and reverse causation—whereby the act of attending for screening, and the accompanying contact with a healthcare provider, itself generated awareness, rather than awareness prompting screening—remains an equally plausible explanation. A related, non-mutually exclusive possibility is that both awareness and screening completion are driven by a woman’s underlying degree of engagement with the healthcare system: women who are more engaged with healthcare in general may be more likely to receive postpartum counselling, to report higher awareness, and to complete screening, independently of any direct causal link between awareness and screening. Because we did not measure a general healthcare-engagement construct, we cannot formally test or adjust for this possibility, and it should be regarded as an unmeasured confounder that our cross-sectional design cannot rule out.

Moving forward, clinical practice must shift away from ‘one-size-fits-all’ strategies toward empathetic, individualized health care, which acknowledges the chaotic realities of postpartum life and ensures that risk awareness is translated into sustained, long-term metabolic follow-up [28]. Consequently, expanding clinical practice beyond initial diagnosis to systematically incorporate structured, recurrent postpartum education would help address current preventive gaps and optimize long-term maternal health in Riyadh.

## 5. Strengths and Limitations

A primary strength of this study lies in its comparative framework, benchmarking risk awareness in women with a self-reported history of gestational diabetes directly against parous women from the same population without self-reported prior GDM. Methodologically, the use of multivariable logistic regression allowed adjustment for current age, educational attainment, and (where relevant) GDM status; however, as detailed below, several other clinically and socioeconomically relevant confounders were not measured, so this adjustment should be regarded as partial rather than comprehensive control of confounding.

Several limitations merit consideration. The cross-sectional design precludes establishing definitive causal pathways between clinical awareness and long-term screening adherence. Risk awareness, postpartum counselling, postpartum screening, and lifestyle modification were all ascertained at, or with respect to, a single point in time, with no dates captured for when each occurred relative to the others; several causal orderings—awareness prompting screening and lifestyle change, or screening and lifestyle change themselves prompting greater self-reported awareness and recollection of counselling—remain equally consistent with the data, and this design cannot adjudicate between them. The postpartum counselling item did not specify a timing window relative to delivery (during pregnancy, immediately postpartum, at a defined 6-week visit, or later follow-up), which further limits interpretation of its relationship to subsequent awareness and screening.

Disseminating the questionnaire exclusively via WhatsApp introduces a specific, directional selection bias: women with lower digital access, lower educational attainment, or less social-media use are plausibly underrepresented, and would independently be expected to show lower T2DM risk awareness. This is consistent with the highly educated skew of the sample (Section 3.1) and would be expected to bias awareness estimates in both groups upward, likely more than it biases the relative GDM-versus-comparison-group contrast that is the focus of this study.

Reliance on self-reported clinical history introduces the potential for exposure misclassification: self-reported prior GDM status is subject to recall bias, and neither GDM diagnosis nor postpartum screening completion was verified against medical or laboratory records—both the primary exposure and the key adherence outcome rest entirely on unverified self-report. Misclassification of GDM status is possible in both directions; some women in the comparison group may have had undiagnosed, undisclosed, or unrecalled GDM, since screening status during pregnancy was not ascertained. Non-differential misclassification of this kind would be expected to attenuate the between-group contrast toward the null, offering a further explanation for the absence of a detected difference in awareness. For the same reason, the between-group comparison of postpartum screening should be read descriptively, since such screening is guideline-indicated only after GDM.

Eligibility for parity rested on a single self-reported item (“Have you ever been pregnant?”), which did not distinguish a pregnancy ending in live birth from one ending in miscarriage, stillbirth, or termination, nor exclude a respondent currently in her first, ongoing pregnancy; no additional questionnaire item, and no external record, confirmed that a given respondent had actually delivered. The term “parous” is therefore used throughout this manuscript to describe the study’s intended target population, but individual respondents’ delivery status was not independently verified, and outcome items presupposing a completed pregnancy (e.g., time since last delivery, postpartum screening uptake) may accordingly carry an unknown degree of misclassification for respondents who had not, in fact, delivered.

Relatedly, the questionnaire did not separately screen for current pregnancy status, so a small number of currently pregnant respondents—whether previously delivered or pregnant for the first time—may have been included alongside postpartum respondents; because several outcome items are not applicable to a currently pregnant woman, this could introduce further non-differential misclassification of the adherence outcomes. Separately, because the questionnaire did not include an independent screening item for age, nationality, or place of residence beyond the eligibility criteria themselves, a small number of ineligible respondents cannot be fully excluded as having been missed rather than genuinely absent from the sample. No item ascertained pre-existing or current T2DM or prediabetes; women with either, who would be expected to show higher diabetes awareness for reasons unconnected to a prior GDM diagnosis, could not be identified or excluded and represent an unmeasured potential confounder of the awareness comparison. These limitations should be addressed by dedicated screening items (confirming live birth specifically, and current pregnancy, prediabetes, and T2DM status) in future survey instruments on this topic.

The questionnaire’s measurement properties were only partly established. Content was reviewed by field experts, including an Associate Professor and Consultant in Obstetrics and Gynecology for the Arabic version, and the instrument was piloted with 10 eligible participants before formal data collection; however, no formal forward-/back-translation procedure was undertaken between the English and Arabic versions, no internal-consistency statistics (e.g., Cronbach’s alpha) were computed for the multi-item awareness and attitude sections, no construct validity analysis (e.g., factor structure) was performed, and test–retest reliability was not assessed. In particular, the primary awareness outcome was not measured using a validated risk-perception or health-literacy instrument; it relied on a single purpose-built item whose psychometric properties beyond expert content review and piloting are unknown, and this should be borne in mind when comparing our awareness estimates with those from studies using validated multi-item instruments. Expert content review is a necessary but not sufficient step toward measurement validity.

Two outcome-definition limitations should also be noted. The postpartum screening item asked only whether blood sugar was checked 6–12 weeks after delivery, without specifying the test used (OGTT, fasting plasma glucose, random glucose, or HbA1c) and using a narrower window than the 4–12-week ADA guidance cited in the Introduction; the outcome should therefore be read as any self-reported postpartum glucose testing within a roughly, but not exactly, guideline-concordant window, rather than as confirmed guideline-specific OGTT completion. The lifestyle-modification outcome was derived from a multi-select item using an “at least one endorsement” coding rule; a stricter definition requiring multiple concurrent behaviour changes could yield different adoption estimates. More broadly, both adherence outcomes are subject to outcome misclassification, since postpartum screening completion and lifestyle modification were both self-reported rather than verified against medical records, laboratory data, or objective behavioural measures, so both recall inaccuracy and social-desirability reporting (a tendency to over-report socially favoured behaviours) could bias the adherence estimates, most plausibly toward over-reporting of adherence in both groups.

The study did not meet its own pre-specified minimum required sample size: 316 women were analysed against a calculated minimum of 384 (and a non-response-inflated target of 461). This shortfall is, on its own, sufficient reason for caution in interpreting every estimate reported here, and especially the primary awareness comparison. The original sample-size calculation was based on a single-proportion formula and therefore did not address the power of the study’s primary between-group comparison. With 88 women in the prior GDM group and 228 controls, the study had 80% power to detect only a difference in awareness of approximately 15 percentage points or greater. The observed difference of 5.4 percentage points (95% CI −5.9 to +16.6) is therefore best interpreted as inconclusive with respect to smaller but potentially clinically meaningful differences, rather than as evidence of equivalence between the groups; a formal equivalence test did not establish equivalence at a ±10 percentage-point margin (*p* = 0.209). Post-hoc power calculations based on observed effects were deliberately not performed, since such calculations are a deterministic function of the obtained *p*-value and provide no additional inferential information; confidence intervals are reported throughout in their place.

A further limitation concerns residual confounding by unmeasured clinical and socioeconomic variables. Although body weight and height were recorded, body mass index was not incorporated into the analytical models, and a subset of anthropometric entries showed unit-conversion inconsistencies that could not be resolved retrospectively; obesity is the principal shared risk factor for both gestational diabetes and subsequent type 2 diabetes, so residual confounding by adiposity cannot be excluded. Family history of type 2 diabetes was ascertained only among participants with prior GDM and could not be adjusted for in whole-sample models. Current pre-existing T2DM and prediabetes were not ascertained and could not be excluded or adjusted for. Time since the most recent delivery was not collected for the whole sample, and time since the GDM-affected pregnancy, although collected for the prior-GDM subgroup, was not entered as a covariate in any regression model.

Gravidity and parity were not collected and could therefore not be examined as potential confounders. No measure of household income, occupational social class, or other socioeconomic status indicator beyond educational attainment and employment status was collected, and no item captured healthcare access directly (e.g., insurance status, distance to or regularity of contact with a primary care provider, or health-system-related barriers to attending screening), even though such access is plausibly associated with both awareness and screening adherence independently of GDM history. Finally, the expected-cell-count threshold used to determine whether the Fisher–Freeman–Halton exact test was required (Section 2.3) was checked for every cross-tabulation reported in the main text and in Table 2 and Table 5, but the more granular sociodemographic cross-tabulations underlying Table 1 were not individually re-verified against this threshold.

Residual confounding by any of these unmeasured or unadjusted factors therefore cannot be excluded, and the adjusted estimates reported in this manuscript should be interpreted as adjusted for current age, educational attainment, and (where stated) GDM status only—not as fully adjusted estimates in a causal sense.

## 6. Conclusions

Awareness of future T2DM risk is widely regarded as an important precondition for engagement with postpartum screening and preventive lifestyle behaviour, though this study did not measure incident diabetes or other long-term metabolic endpoints and cannot itself demonstrate that greater awareness prevents progression to T2DM. To meaningfully improve postpartum diabetes prevention in Riyadh, routine clinical practice should expand beyond the initial detection of GDM to include structured postpartum education alongside explicit transition protocols between obstetricians and primary care providers, addressing current gaps in postpartum care.

## Figures and Tables

**Table 3:** Multivariable Logistic Regression Model of High T2DM Risk Awareness.

Predictor	Adjusted OR	95% CI	*p*-Value
Prior GDM diagnosis	1.02	0.57–1.82	0.936
Age ≥ 45 years	2.41	1.41–4.11	0.001
Bachelor’s degree or above	1.22	0.69–2.14	0.495

Note. After adjustment, prior GDM showed no independent association with risk awareness, whereas an older current age remained a strong and significant correlate.

**Table 4:** Multivariable Logistic Regression of Self-Reported Postpartum Glucose Screening (dependent variable: “Yes” to “Did you get your blood sugar checked 6 to 12 weeks after you delivered your baby?”, coded 1 = yes vs. 0 = no/don’t remember/not advised; Section 2.2).

Predictor Variable	Adjusted OR (aOR)	95% CI	*p*-Value
High risk awareness (ref: low/moderate awareness)	3.57	2.04–6.23	<0.001
Prior GDM diagnosis (ref: no self-reported prior GDM)	2.18	1.27–3.74	0.005
Age ≥ 35 years (ref: under 35 years)	0.98	0.56–1.73	0.955
Higher education (BA+) (ref: below bachelor’s degree)	1.27	0.73–2.20	0.395

Note. *n* = 316; 124 events; 31 events per variable. Hosmer–Lemeshow χ^2^ = 6.17, df = 6, *p* = 0.405; C-statistic = 0.690; VIF ranged from 1.01 to 1.08. This model uses a ≥35-year age dichotomisation to capture clinically relevant T2DM risk among women of reproductive age, which differs from the ≥45-year cutoff used for current age classification in Table 1, Table 2 and Table 3.

## Data Availability

The raw data supporting the conclusions of this article will be made available by the authors, without undue reservation, upon reasonable request to the corresponding author. Specifically, this includes de-identified participant-level data, the English- and Arabic-language questionnaires (Supplementary Files S1 and S2), the variable codebook, and the statistical analysis syntax used to produce the results reported in this manuscript.

## Supplementary Materials

The following supporting information can be downloaded at: https://www.mdpi.com/article/10.3390/healthcare14162572/s1, File S1: English-language version of the study questionnaire; File S2: Arabic-language version of the study questionnaire (item-for-item equivalent to S1), used to assess T2DM risk awareness, postpartum counselling and screening history, and lifestyle modification, as described in Section 2.2.
