# Supplementary material for: Awareness of Future Type 2 Diabetes Risk Among Parous Women With and Without Self-Reported Prior Gestational Diabetes in Riyadh, Saudi Arabia: A Questionnaire-Based Cross-Sectional Survey
Source: Healthcare (Basel). 2026 Aug 17;14(16):2572. doi: 10.3390/healthcare14162572 (PMC13512273; doi:10.3390/healthcare14162572)
Supplement: Supplementary file 1 [file healthcare-14-02572-s001.zip › File_S1.pdf]

Questionnaire: Awareness of Type 2 Diabetes Risk: A Comparison Between Women With and Without Prior Gestational Diabetes in Riyadh.
Introduction and Informed Consent

*Dear **participant,***
Thank you for taking the time to participate in this study. The purpose of this research is to compare the knowledge regarding the future risk of developing Type 2 Diabetes among women in Riyadh, both with and without a history of Gestational Diabetes.
Your participation is completely voluntary, and all your responses will remain strictly anonymous and confidential. The survey will take approximately **3-5 minutes** to complete.

- 1. Do you consent to participate in this study?
    - [ ] Yes *(Proceed to survey)*
    - [ ] No *(End survey)*
- 2. Have you ever been pregnant?
    - [ ] Yes *(Proceed to survey)*
    - [ ] No *(End survey)*

Section 1: Socio-Demographic Information
This section helps us understand the background of our participants.

3. What is your current age?
- [ ] 18–24 years
- [ ] 25–34 years
- [ ] 35–44 years
- [ ] 45 years or older

4. What is your highest level of education?
- [ ] High school or below
- [ ] Diploma
- [ ] Bachelor’s Degree
- [ ] Postgraduate Degree (Master's/PhD)

5. What is your current employment status?
- [ ] Employed (Private sector /Government sector)
- [ ] Homemaker / Unemployed
- [ ] Student

6. What region/area of Riyadh do you currently live in?
- [ ] North Riyadh
- [ ] South Riyadh
- [ ] East Riyadh
- [ ] West Riyadh
- [ ] Central Riyadh

7. Anthropometric Measurements (Optional)
- Current Weight (kg): ______
- Height (cm): ______

Section 2: Obstetric and Medical History

This section gathers information about your pregnancy and medical background.

8. Have you been diagnosed with Gestational Diabetes Mellitus (GDM) by a physician?

- [ ] Yes *(Continue the section)*
- [ ] No *(Skip this section — proceed to Question 13)*

9. How long has it been since your last pregnancy where you were diagnosed with Gestational Diabetes (GDM)?

- [ ] Less than 1 year
- [ ] 1 to 3 years
- [ ] 4 to 5 years
- [ ] More than 5 years

10. How was Gestational Diabetes managed during that pregnancy?

- [ ] Dietary modifications and lifestyle changes only
- [ ] Oral medication (e.g., metformin) or insulin injections
- [ ] I don't remember

11. Were you advised to undergo regular diabetes screening after delivery?

- [ ] Yes, and follow-up appointments were scheduled
- [ ] Yes, but only through verbal advice
- [ ] No, I was not advised

12. Do you have a family history of Type 2 Diabetes? (Parents, siblings, or grandparents)

- [ ] Yes
- [ ] No
- [ ] I don't know

Section 3: Knowledge of Future Type 2 Diabetes Risk

Please answer what you currently know or believe. There are no "wrong" answers—we just want to understand your current awareness.

13. Are you aware that having Gestational Diabetes increases a woman's risk of developing Type 2 Diabetes later in life?

- [ ] Yes, I am fully aware
- [ ] I have heard about it, but I'm not sure
- [ ] No, I was not aware

14. In your opinion, how long after delivery is a woman with a history of GDM most at risk for developing Type 2 Diabetes?

- [ ] Within the first 5 years
- [ ] More than 5 years
- [ ] The risk remains lifelong
- [ ] I don't know

15. Which of the following factors do you think further increases your risk of developing Type 2 Diabetes? <u>(Select all that apply)</u>

- [ ] Being overweight or obese

- [ ] Lack of physical activity
- [ ] Unhealthy diet
- [ ] Having a family history of diabetes
- [ ] None of the above

16. What is your primary source of information about diabetes risk?

- [ ] Healthcare provider (physician/nurse)
- [ ] Social media
- [ ] Family and friends
- [ ] Personal online research

Section 4: Attitude and Risk Perception

Please indicate how much you agree or disagree with the following statements.

17. "I believe I am personally at a high risk of developing Type 2 Diabetes in the future."

- [ ] Strongly Agree | [ ] Agree | [ ] Neutral | [ ] Disagree | [ ] Strongly Disagree

18. "I believe that Type 2 Diabetes can be delayed or prevented through lifestyle changes."

- [ ] Strongly Agree | [ ] Agree | [ ] Neutral | [ ] Disagree | [ ] Strongly Disagree

---

Section 5: Preventive Practices and Screening

This section asks about your actions since your pregnancy.

19. Did you get your blood sugar checked 6 to 12 weeks after you delivered your baby?

- [ ] Yes
- [ ] No
- [ ] I don't remember
- [ ] I wasn't asked to do that.

20. How often do you currently check your blood sugar levels?

- [ ] Every 1–3 years (as recommended)
- [ ] Only when I feel unwell
- [ ] Rarely or never

21. What changes have you made to prevent diabetes? (Select all that apply.)

- [ ] Improved my diet
- [ ] Increased my physical activity/exercise
- [ ] Actively managing my weight
- [ ] No changes made

22. If you do not follow a healthy lifestyle, what is the main barrier?

- [ ] Lack of time
- [ ] Financial cost (e.g., healthy foods or gym memberships)
- [ ] Lack of social support from family
- [ ] Not convinced that lifestyle changes are beneficial
