# Supplementary material for: Awareness of Future Type 2 Diabetes Risk Among Parous Women With and Without Self-Reported Prior Gestational Diabetes in Riyadh, Saudi Arabia: A Questionnaire-Based Cross-Sectional Survey
Source: Healthcare (Basel). 2026 Aug 17;14(16):2572. doi: 10.3390/healthcare14162572 (PMC13512273; doi:10.3390/healthcare14162572)
Supplement: Supplementary file 1 [file healthcare-14-02572-s001.zip › File_S2.pdf]

استبيان: الوعي بمخاطر الإصابة بداء السكري من النوع الثاني: مقارنة بين النساء المصابات وغير المصابات بسكري الحمل في الرياض.

المقدمة وأخذ الموافقة

عزيزتي **المشاركة**، نشكرك على تخصيص الوقت للمشاركة في هذه الدراسة. يهدف هذا البحث إلى **مقارنة المعرفة المتعلقة بخطر الإصابة بداء السكري من النوع الثاني في المستقبل بين النساء في الرياض، سواء كان لديهن تاريخ من سكري الحمل أم لا.** مشاركتك تطوعية تمامًا، **كما أن جميع إجاباتك مجهولة المصدر (لا توجد أي معلومات شخصية).** سيستغرق الاستبيان **5-3 دقائق** لإكماله.

- **1. هل توافقين على المشاركة في هذه الدراسة؟**

[ ] نعم *(المتابعة إلى اكمال الاستبانة)*
[ ] لا *(إنهاء الاستبانة مباشرة)*

2. هل سبق لك الحمل؟

[ ] نعم *(المتابعة في اكمال الاستبانة)*
[ ] لا *(إنهاء الاستبانة مباشرة)*

القسم 1: المعلومات الاجتماعية والديموغرافية

يساعدنا هذا القسم على فهم خلفية المشاركات.

3. ما هو عمرك الحالي؟

[ ] 18-24 سنة
[ ] 25-34 سنة
[ ] 35-44 سنة
[ ] 45 سنة أو أكثر

4. ما هو أعلى مستوى تعليمي لك؟

[ ] ثانوية عامة أو أقل
[ ] دبلوم
[ ] درجة البكالوريوس
[ ] دراسات عليا (ماجستير/دكتوراه)

5. ما هي حالتك الوظيفية الحالية؟

[ ] موظفة (قطاع عام/ قطاع خاص)
[ ] ربة منزل / غير موظفة
[ ] طالبة

6. في أي منطقة من الرياض تعيشين حاليًا؟

[ ] شرق الرياض
[ ] شمال الرياض
[ ] جنوب الرياض
[ ] غرب الرياض
[ ] وسط الرياض

7. القياسات الجسمانية (اختياري)؟

- الوزن الحالي (بالكيلوجرام): ______
- الطول (بالسنتيمتر): ______

القسم 2: التاريخ الطبي وتاريخ الولادة
يجمع هذا القسم معلومات حول حملك وخلفيتك الطبية.

8. هل تم تشخيصك بسكري الحمل (GDM) من قبل طبيب؟

[ ] نعم *(المتابعة في القسم الحالي)*
[ ] لا *(تخطي القسم الحالي - الذهاب مباشرة للسؤال ١٣)*

9. كم مضى من الوقت منذ آخر حمل تم تشخيصك فيه بسكري الحمل (GDM)؟

[ ] أقل من سنة
[ ] من 1 إلى 3 سنوات
[ ] من 4 إلى 5 سنوات
[ ] أكثر من 5 سنوات

10. كيف تمت إدارة سكري الحمل في ذلك الحمل؟

[ ] النظام الغذائي ونمط الحياة فقط
[ ] أدوية فموية (مثل الميتفورمين) أو حقن الأنسولين
[ ] لا أتذكر

11.هل تم إخباركِ بضرورة إجراء فحص دوري للسكري بعد الولادة؟

[ ] **نعم، وتم تحديد المواعيد.**
[ ] **نعم، ولكن بشكل شفهي فقط.**
[ ] **لا، لم يتم إخباري.**

12. هل لديك تاريخ عائلي لمرض السكري من النوع 2؟ (الوالدان، الإخوة والأخوات، أو الأجداد)

[ ] نعم
[ ] لا
[ ] لا أعرف

القسم 3: المعرفة بمخاطر الإصابة بمرض السكري من النوع 2 في المستقبل
يرجى الإجابة بما تعرفينه أو تعتقدينه حاليًا. لا توجد إجابات "خاطئة" - نحن نريد فقط فهم وعيك الحالي.

13. هل تدركين أن سكري الحمل يزيد من خطر الإصابة بالسكري النوع الثاني لاحقاً؟

[ ] نعم، أنا على دراية تامة
[ ] سمعت عن ذلك ولست متأكدة.
[ ] لا، لم أكن على دراية

14. في رأيك، بعد كم من الوقت من الولادة تكون المرأة التي لديها تاريخ مع سكري الحمل أكثر عرضة لخطر الإصابة بمرض السكري من النوع 2؟

[ ] خلال السنوات الخمس الأولى
[ ] بعد أكثر من 5 سنوات

[ ] الخطر مستمر مدى الحياة.
[ ] لا أعرف

15. أي من العوامل التالية تعتقدين أنها تزيد من خطر إصابتك بمرض السكري من النوع 2؟ (حددي كل ما ينطبق)

[ ] زيادة الوزن أو السمنة
[ ] قلة النشاط البدني
[ ] النظام الغذائي غير الصحي
[ ] وجود تاريخ عائلي للإصابة بمرض السكري
[ ] لا شيء مما سبق

16. ما هو مصدرك الرئيسي للمعلومات حول مخاطر السكري؟

[ ] مقدم الرعاية الصحية (طبيب/ممرض).
[ ] وسائل التواصل الاجتماعي.
[ ] الأهل والأصدقاء.
[ ] البحث الشخصي عبر الإنترنت.

القسم 4: الموقف وإدراك المخاطر
يرجى الإشارة إلى مدى موافقتك أو عدم موافقتك على العبارات التالية.

17. "أعتقد أنني شخصياً معرضة لخطر كبير للإصابة بمرض السكري من النوع 2 في المستقبل."
[ ] أوافق بشدة | [ ] أوافق | [ ] محايد | [ ] لا أوافق | [ ] لا أوافق بشدة

18. "أعتقد أنه يمكن تأخير أو الوقاية من مرض السكري من النوع 2 من خلال تغييرات في نمط الحياة."
[ ] أوافق بشدة | [ ] أوافق | [ ] محايد | [ ] لا أوافق | [ ] لا أوافق بشدة

القسم 5: الممارسات الوقائية والفحص
يسأل هذا القسم عن إجراءاتك منذ حملك.

19. هل قمت بفحص مستوى السكر في الدم بعد 6 إلى 12 أسبوعًا من ولادة طفلك؟

[ ] نعم
[ ] لا
[ ] لا أتذكر
[ ] لم يُطلب مني ذلك

20. كم مرة تقومين حاليًا بفحص مستويات السكر في الدم؟

[ ] كل 1-3 سنوات (كما هو موصى به)
[ ] فقط عند الشعور بتوعك.
[ ] نادراً أو أبداً

21. ما هي التغييرات التي قمتِ بها للوقاية من السكري؟ (حددي كل ما ينطبق)

[ ] تحسين نظامي الغذائي
[ ] زيادة نشاطي البدني/ ممارسة الرياضة
[ ] إدارة الوزن بنشاط
[ ] لم أقم بأي تغييرات

22.في حال عدم التزامك بنمط حياة صحي، ما هو العائق الرئيسي؟

[ ] ضيق الوقت.
[ ] التكلفة المادية (للأغذية الصحية أو النوادي)
[ ] نقص الدعم الاجتماعي من العائلة.
[ ] عدم القناعة بجدوى التغيير.
